# 3D convolutional neural networks applied to CT angiography in the detection of acute ischemic stroke

**DOI:** 10.1186/s41747-019-0085-6

**Published:** 2019-02-13

**Authors:** Olli Öman, Teemu Mäkelä, Eero Salli, Sauli Savolainen, Marko Kangasniemi

**Affiliations:** 1 HUS Medical Imaging Center, Radiology, University of Helsinki and Helsinki University Hospital, P.O. Box 340 (Haartmaninkatu 4), FI-00290 Helsinki, Finland; 2 Department of Physics, University of Helsinki, P.O. Box 64, FI-00014 Helsinki, Finland

**Keywords:** Computed tomography angiography, Convolutional neuralnetwork, Machine learning, Neural networks (computer), Stroke

## Abstract

**Background:**

The aim of this study was to investigate the feasibility of ischemic stroke detection from computed tomography angiography source images (CTA-SI) using three-dimensional convolutional neural networks.

**Methods:**

CTA-SI of 60 patients with a suspected acute ischemic stroke of the middle cerebral artery were randomly selected for this study; 30 patients were used in the neural network training, and the subsequent testing was performed using the remaining 30 patients. The training and testing were based on manually segmented lesions. Cerebral hemispheric comparison CTA and non-contrast computed tomography (NCCT) were studied as additional input features.

**Results:**

All ischemic lesions in the testing data were correctly lateralized, and a high correspondence to manual segmentations was achieved. Patients with a diagnosed stroke had clinically relevant regions labeled infarcted with a 0.93 sensitivity and 0.82 specificity. The highest achieved voxel-wise area under receiver operating characteristic curve was 0.93, and the highest Dice similarity coefficient was 0.61. When cerebral hemispheric comparison was used as an input feature, the algorithm performance improved. Only a slight effect was seen when NCCT was included.

**Conclusion:**

The results support the hypothesis that an acute ischemic stroke lesion can be detected with 3D convolutional neural network-based software from CTA-SI. Utilizing information from the contralateral hemisphere appears to be beneficial for reducing false positive findings.

## Key points

- This is the first study applying three-dimensional convolutional neural networks (3D CNN) to computed tomography angiography (CTA) source images for ischemic stroke detection
- Stroke detection was improved when cerebral hemispheric comparison and non-contrast computed tomography (NCCT) were included in the CNN analysis.
- High sensitivity and specificity in the detection of stroke lesions was achieved

## Background

An acute ischemic stroke is caused by thrombotic or embolic occlusion of a cerebral artery. Occlusion of the proximal cerebral artery causes a deep ischemia resulting in a collapse of cellular energetics. This is followed by a necrotic cell death in few minutes. The infarct core is surrounded by a partial area of ischemia, penumbra, where neurons will die within hours. Accurate identification of this “tissue at risk” could be used to identify patients who would benefit most from treatment [1]. In a large ischemic stroke, the extent of damage will increase during the following days. In an extreme case, the mass effect coupled with the tissue damage leads to an increased intracranial pressure and a loss of cerebral blood circulation.

The restoration of blood flow to the tissue can be achieved by opening the embolic artery. An essential aim of acute treatment of brain infarction is to restrict the size of the infarct by a rapid and permanent recanalization of the obstructed artery. Primary imaging methods for the acute ischemic stroke are non-contrast computed tomography (NCCT), CT perfusion (CTP), and CT angiography (CTA). The advantage of CT-based methods is that they are rapid and more widely available than MRI in the emergency setting.

The NCCT imaging must be performed as soon as possible after the stroke code has been activated [2]. NCCT is highly sensitive for the depiction of hemorrhagic lesions [3] or other NCCT visible mimics of the stroke. The crucial role of NCCT is also the detection of ischemic signs of established ischemic lesion. The main NCCT finding is a cortical-subcortical hypoattenuating area within a vascular territory. It is, however, well-known that NCCT has a relatively low sensitivity in the first 24 h, especially within the limited (3–6 h) time window for thrombolytic treatment [4].

The extension of the acute infarct is typically estimated from CT perfusion parameters (mean transit time, cerebral blood volume, and cerebral blood flow). In CTP, alterations in cerebral blood flow and volume can be seen in the acute stroke patients. However, many multi-detector scanners still offer limited coverage for CTP of the brain which is often stated as a drawback of CTP. Newer 256-slice scanners can provide whole-brain coverage, and in the next few years this coverage will most likely be widely available [4]. CTP implies a relatively high radiation exposure and coarser resolution and thicker slices when compared, for example, to CTA.

Using CTA, it is possible to demonstrate the anatomy of the aortic arch, carotid, and cerebral arteries, the presence of stenosis or thrombus of carotid or cerebral arteries. In addition, an impression of the functioning collateral network is obtained. Hypoattenuation in CTA source images (CTA-SI) showing a lack of enhancement provides an estimate of the cerebral blood flow reduction, whereas the NCCT allows to assess changes in brain tissue water content. In comparison, CTP is performed by monitoring only the first pass of an iodinated contrast agent bolus through the cerebral circulation [5]. Interestingly, CTA-SI can provide another way, complementary to CTP, to evaluate the size of acute infarct [6, 7], even though this method is not typically used in clinical practice. CTA detects the regions of the brain with hypoattenuation due to long delays in contrast arrival to the ischemic brain tissue. It has been discussed in several studies (*e.g.,* [8]) about CTA correlating more with a CTP cerebral blood flow map (penumbra and core) than with the corresponding cerebral blood volume map [8].

Among the CT techniques, CTA has multiple advantages. It is highly specific and more sensitive than NCCT in the detection of early irreversible ischemia [9]. In addition, it provides the possibility to evaluate the whole brain vasculature which is not available with CT perfusion. The clinical outcome following the recanalization has been discussed in other studies, and it is believed to be strongly dependent on the extent of the pretreatment infarct lesion volume [10]. The total stroke lesion volume can be used as a predictor of treatment efficacy. A pre-treatment infarct volume lower than 70 cm^3^ can be set as threshold for predicting a good clinical response to reperfusion [11].

Applying artificial intelligence to stroke sign detection can potentially aid in diagnosis or predicting prognosis [12]. One of the motivations in applying machine learning and deep learning methods to medical images is the automatic extraction of non-trivial and non-linear features from the imaging data. These features can then be applied for example in tissue classification or lesion volumetry. If the imaging data are inherently three-dimensional (3D), it is natural to use methods based on 3D features. One such AI algorithm family is 3D convolutional neural networks (CNNs). They increase computational costs and hardware requirements compared to their two-dimensional counterparts. However, parallel processing, especially utilization of graphics processing units, as well as the recent advancement in deep learning algorithms, have allowed 3D CNN-based software tools to take further steps toward clinical deployment.

CNNs and their application in medical imaging have been discussed in length by other researchers [13–15]. A review of AI application areas in stroke imaging have been written by Lee et al. [12]. Recently, Lisowska et al. [16] studied the detection of dense vessels and of ischemia from NCCT images using CNNs.

An automatic tool to detect infarcted regions from brain CTA-SI could aid in diagnosis and evaluate the infarct extent or volume.

This study had two aims: (1) to investigate if using a 3D CNN is feasible for detecting and segmenting hypoattenuated regions in CTA-SI of stroke patients; and (2) to investigate if analysis performance can be improved by including NCCT and information from contralateral hemisphere as inputs to the neural network. The training and evaluation of the networks were based on manual segmentations of the hypoattenuated regions. This is the first study investigating the application of 3D CNN on CTA-SI for acute ischemic stroke lesion detection.

## Methods

### Subjects and imaging parameters

The clinical and imaging findings of the consecutive stroke suspected cases of the middle cerebral artery or/and internal carotid artery occlusion presented to Helsinki University Hospital between November 2016 and December 2016 were retrospectively reviewed, and 60 randomly selected patients were included in the study. All of them had undergone an identical CT protocol. Thirty patients had been diagnosed by neurologists with an acute ischemic stroke (group A). The CTA and CTP of the other 30 patients showed no signs of a stroke (group B). Both groups were randomly selected from the corresponding patient populations (the group A was limited to the patients with unilateral ischemic strokes of the middle cerebral artery). Inclusion criteria were (1) stroke code activated; (2) admission stroke protocol imaging performed using fast CTA-SI acquisition protocol; and (3) documented middle cerebral artery or/and internal carotid artery occlusion on CTA-SI.

In the group A, the median (range) National Health Stroke Scale (NIHSS) was 17 (3–27). Disease burden in the group A consisted of total of 15 patients which were earlier diagnosed with a cardiovascular disease (atrial fibrillation and/or coronary artery disease), four had a type 2 diabetes, four had a diagnosis of breast cancer, and two had a diagnosis of chronic obstructive pulmonary diagnosis. The rest of the group A were disease-free before the acute stroke. In the group B, the patients presented with symptoms suggestive of stroke (focal neurological symptoms with sudden onset). The group B was randomly selected from patients imaged with the stroke protocol who did not have a diagnosis of stroke. Seventeen patients in the group B underwent a follow-up brain MRI in the following 48 h from stroke code activation without stroke diagnosis. The rest of the stroke-negative group B were domiciliated by the neurologists during the next 24 h without the follow-up scan and without diagnosis of stroke.

In the group A, the number of patients with occluded middle cerebral artery segments (M1–4) and internal carotid artery (ICA) were M1 (*n* = 16); M2 (*n* = 5); M3 (*n* = 1); M4 (*n* = 1); ICA (*n* = 4); ICA + M1 (*n* = 1); and ICA + M2: (*n* = 2). The symptoms had begun ≤ 1 h (*n* = 8), 1–2 h (*n* = 7), 2-3 h (*n* = 2), and ≥ 3 h (*n* = 12) prior to the CT in the group A. The group A consisted of 15 males and 15 females while the group B consisted of 14 male and 16 female patients. The median (min-max) ages were 73 (47–82) years and 63 (26–91) years in the groups A and B, respectively. All the patients were imaged using a Somatom Definition Edge (Siemens Healthineers, Erlangen, Germany) 128-slice CT scanner, with the same protocol described in Table 1. CTA scanning range included the carotid arteries. NCCT and CTP were limited to the intracranial range. Due to the retrospective nature of the study, only transversal reconstructions with slice thicknesses/increments of 0.75/0.5 mm (CTA) and 4/4 mm (NCCT) were available for the analysis described below. Due to the manually adjusted field-of-views, nominal in-plane resolutions were from 0.32 × 0.32 mm^2^ to 0.55 × 0.55 mm^2^ (fixed 512 × 512 matrix). All data were anonymized and stored on a server running the Extensible Neuroimaging Archive Toolkit, or XNAT, version 1.1.6 [17].

**Table 1:** Stroke scan protocol. Scout images and test bolus details are not reported

	Non-contrast CT	CT perfusion	CT angiography
Tube voltage (kVp)	120	80	120
Reference current time (*Q*_ref_ mAs)	273	120	150
Reconstruction kernel	J45 s	H20f	I30f
Pitch	0.55	0.5	1.3
Contrast agent administration	–	45 mL (6 mL/s)	50 mL (5 mL/s)
Contrast agent timing	–	6 s delay	Test bolus time to peak + 12 s
Dose length product (mGy · cm)	540	1430	370

*CT* computed tomography, *Q*_*ref*_ quality reference effective tube current-time product (used by Siemens Healthineers)

### Preprocessing

**Fig. 1:**
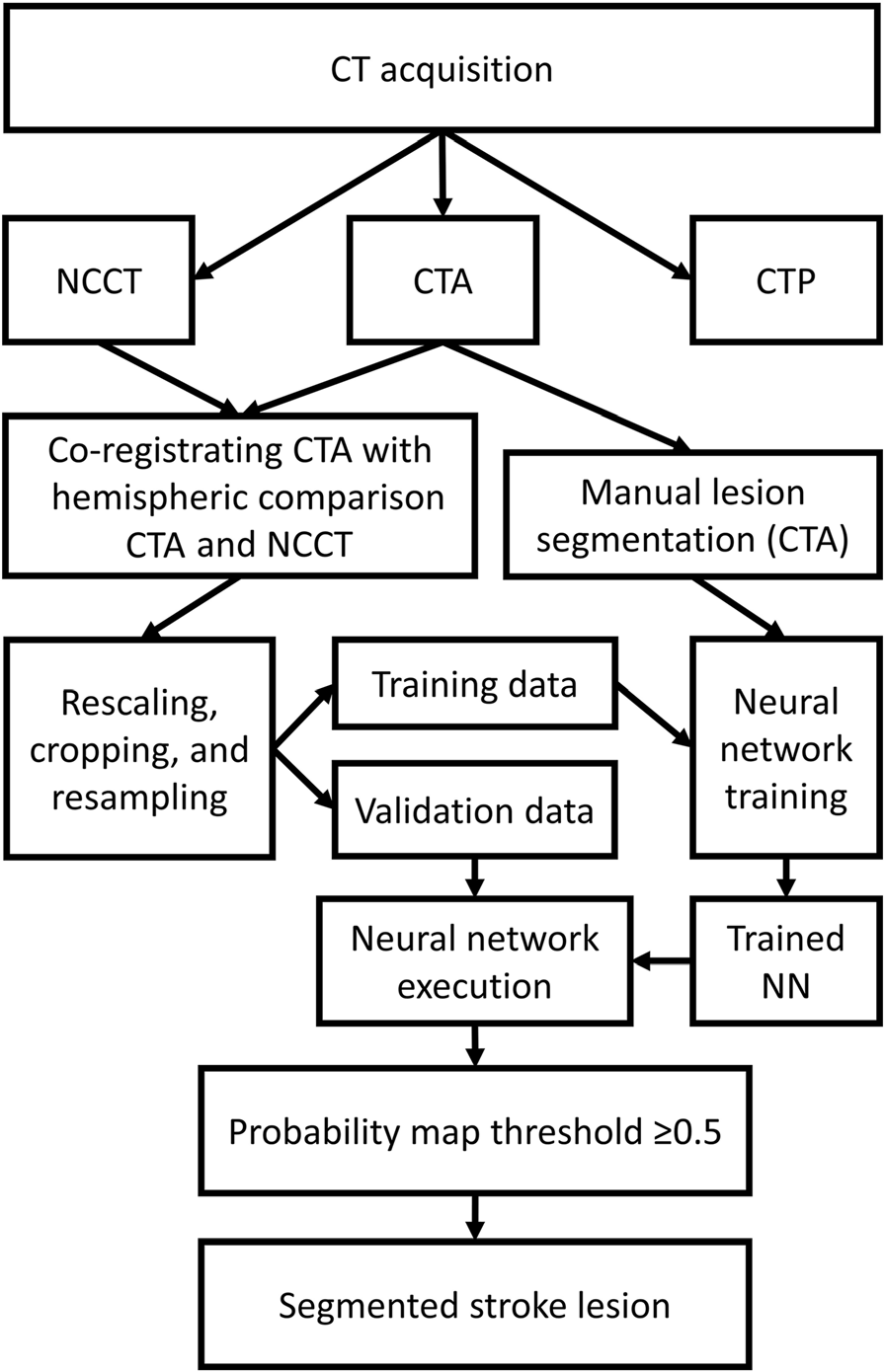
The data processing workflow. CT, computed tomography; NCCT, non-contrast CT; CTA, CT angiography; CTP, CT perfusion; NN, neural network

A senior neuroradiologist and a radiologist, with over 20 and over 5 years of experience, respectively, segmented the infarct regions on the CTA-SI for the 30 stroke-positive patients (group A), in consensus, only including the visible hypoattenuated regions. No manual delineation was done for the group B. The overview of image processing workflow is presented in Fig. 1.

The CNN implementation had three requirements for the input data. Firstly, patient’s CT and CTA image series were cropped to equal image volume dimensions. Secondly, all the data over all the patients needed to have the same image resolution. Thirdly, the data was recommended to be zero mean, and unit variance normalized to aid the CNN to converge.

The CTA volumes (which included carotid arteries and neck region) were cropped to include only the brain region. This was done to decrease memory requirements. To include information from the contralateral hemisphere, an additional set of images was created by flipping the left and right sides of the original CTA-SI, hereafter called *hemispheric comparison volume*. This allowed for the inclusion of the information from the approximate contralateral anatomical regions for the CNN training and evaluation. The flipped images and the NCCT images were then matched to the original images using rigid registration. The image co-registrations were done using BRAINSFit tool [18]. Approximate intracranial spaces were identified using an in-house MATLAB (MathWorks, Inc., Natick, MA, USA) script. Then, the data were scaled to zero mean and unit variance in the brain regions. Finally, all the volumes across the data set were resampled to 0.5 × 0.5 × 0.5 mm isotropic resolution. Convert 3D (part of ITK-SNAP toolkit [19]) command-line tool was used for rescaling and resampling. The consistent voxel size across the data and the intensity shift and rescaling were required for the CNN framework in use. Within each subject, the volumes, including the manual lesion delineations via the nearest neighbor resampling, were in the same image space. All the pre-processing tools had command-line interfaces allowing easy automation and preprocessing multiple subjects in parallel.

All images were eventually verified visually to confirm the successful registrations. 3D Slicer image processing and visualization platform [20] was used in the manual segmentation and the visual verification of the preprocessing outcomes.

### Convolutional neural network

DeepMedic [21], an efficient 3D CNN available as a free open-source implementation based on Theano library [22], was used in the machine learning of the infarct features. The multi-scale network was 11-layers deep with a batch size of 10, L1 regularization 10^−6^ and L2 regularization 10^−4^. Root mean square propagation optimizer (ρ = 0.9, ε = 10^− 4^) was used with Nesterov momentum value 0.6. Training was done over 35 epochs with 15 sub-epochs each. Initial learning rate was set to 0.001 and halved at eight predefined epochs. To remove left-right directional dependence from the trained model, during CNN training, the data were augmented by reflecting all the images along sagittal axis. This step mirrors all the images in unison and is therefore not related to the hemispheric difference volume mentioned above.

The patient groups (A and B) were partitioned randomly into two sets: training set (15 from A + 15 from B) and testing set (15 + 15). The testing set was separate from the training phase and was used only in the reporting of the results. An intracranial mask was used to limit the investigative volume. The manually drawn stroke volumes were considered as ground truths. The output consisted of a single label, *i.e.,* the estimated infarct lesion.

Without changing the data set partitioning or network parameters, three different training runs were performed: (1) CTA as a single input; (2) CTA and left-right flipped CTA for cerebral hemispheric comparison as two inputs; and (3) CTA, flipped CTA for cerebral hemispheric comparison, and NCCT as three inputs. All three CNN outputs were evaluated with the same testing data. The CNNs produced confidence estimates (or *probability maps*) valued from zero to one for the infarcted regions. In addition to voxel-wise segmentation, clinically relevant anatomical regions were recorded as stroke-positive or stroke-negative. Standard anatomical regions used by the Alberta stroke program early CT scoring system (ASPECTS) were chosen as these regions. If a CNN produced even a single positive voxel in the ASPECTS anatomical region, the region was defined as stroke-positive.

All preprocessing, training, and testing were done on a Linux (Debian 8.5) workstation with Intel Xeon E5-2687 W v4 3.00 GHz processor, 64GB RAM and NVIDIA Quadro M6000 24GB graphics processing unit.

### Evaluation methods

The CNN performance was evaluated against the expert segmentation by two approaches, *i.e.,* by anatomical regions visually and in a volumetric voxel-wise manner. The predefined anatomical regions according to the ASPECTS [23] were six segments of middle cerebral artery (M1–M6); insular ribbon (I); nucleus lentiform (L); caudate (C); and internal capsule (IC). These were visually marked as stroke-positive or stroke-negative in the ground truth data and in the three CNN outputs. This approach was chosen to emphasize clinically relevant brain subregions in the evaluation. Secondly, full-brain voxel-wise overlaps between the outcomes and ground truth were compared by calculating Dice similarity coefficients (DSC) and infarct volumes. On all CNN outputs, a conservative threshold probability value of 0.5 was used for labeling voxels as positive. Receiver operating characteristic (ROC) curves were calculated for the voxel-wise overlap by varying the threshold value, and the areas under the curves (AUC) were calculated. The extent and location of the false positive outputs of the CNNs were re-investigated visually by both radiologists. This was done to estimate the possible reasons for false positives. ASPECTS scorings for manual and CNN segmentations of the CTA-SI were calculated for the 30 group A test patients, to concretize the individual differences between the three CNN options.

CNN training is a computationally demanding process, whereas creating ground truths by expert segmentation is often expensive, tedious, and time consuming. To give an oversight of these expenses, CNN training durations were recorded, and one subject was chosen from the group A for which the manual segmentation, automatic preprocessing run, and CNN execution times were recorded.

## Results

**Fig. 2:**
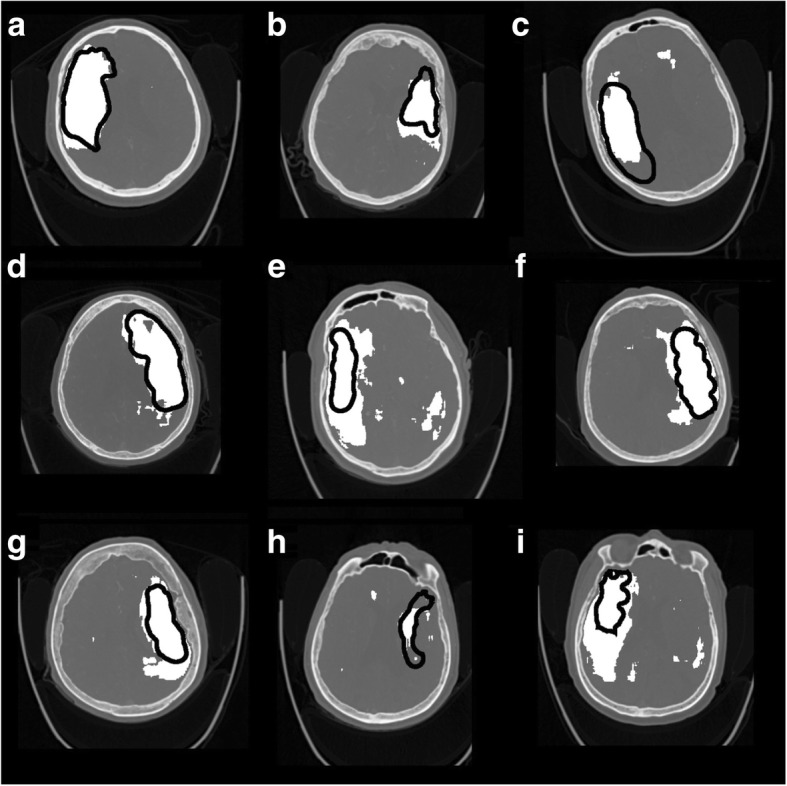
Representative slices of nine (**a**-**i**) of the infarct positive test cases. 3D CNN output with CTA + cerebral hemispheric comparison + NCCT features are shown in white, and the infarcted lesion drawn manually is indicated with black perimeters

**Fig. 3:**
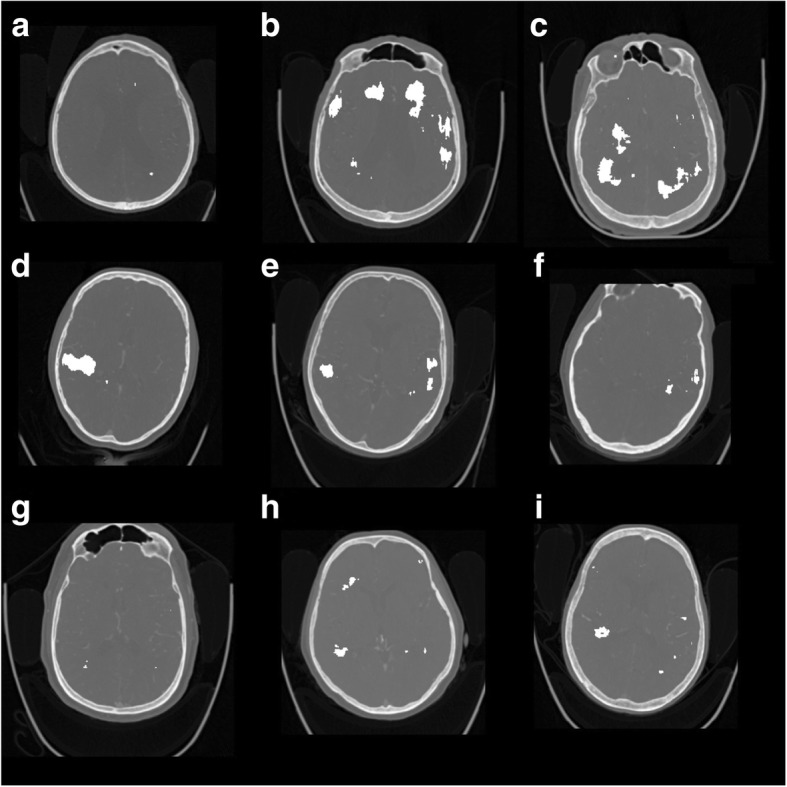
Representative slices of nine (**a**-**i**) of the infarct negative test cases. 3D CNN (false positive) output with CTA + cerebral hemispheric comparison + NCCT features are shown in white

Representative CTA-SI slices with manual and one of the CNN segmentations are shown in Fig. 2 for nine patients of group A. The CNN false positives are shown in Fig. 3 for nine patients of group B. Ten individual anatomical regions (according to the ASPECTS, left and right hemisphere) were manually labeled as positive or negative in the three CNN outputs (Table 2). The manual segmentations were considered as the ground truths. The Dice similarity coefficient (DSC) was calculated from the regions’ true or false labeling (see Table 2).

**Table 2:** Total number of infarct positive brain regions in 15 test subjects in group A and 15 non-infarcted test subjects in group B compared with radiologist evaluation

CNN features	Group A							Group B	Group A + group B
	TN	TP	FN	FP	DSC	Sensitivity	Specificity	FP	DSC
CTA	78	99	6	117	0.62	0.94	0.40	92	0.48
CTA + hemispheric comparison	158	98	7	37	0.82	0.93	0.81	46	0.69
CTA + hemispheric comparison + non-contrast CT	159	98	7	36	0.82	0.93	0.82	34	0.72

The number of true negative (TN), true positive (TP), false negative (FN), and false positive (FP) regions were used to calculate sensitivity specificity and Dice similarity coefficient (DSC). Convolution neural networks CNNs were evaluated for computed tomography angiography (CTA) alone, for CTA plus hemispheric comparison, and for CTA plus hemispheric comparison plus non-contrast CT. For the regions (M1–6, I, L, C, IC on both hemispheres), see text

**Fig. 4:**
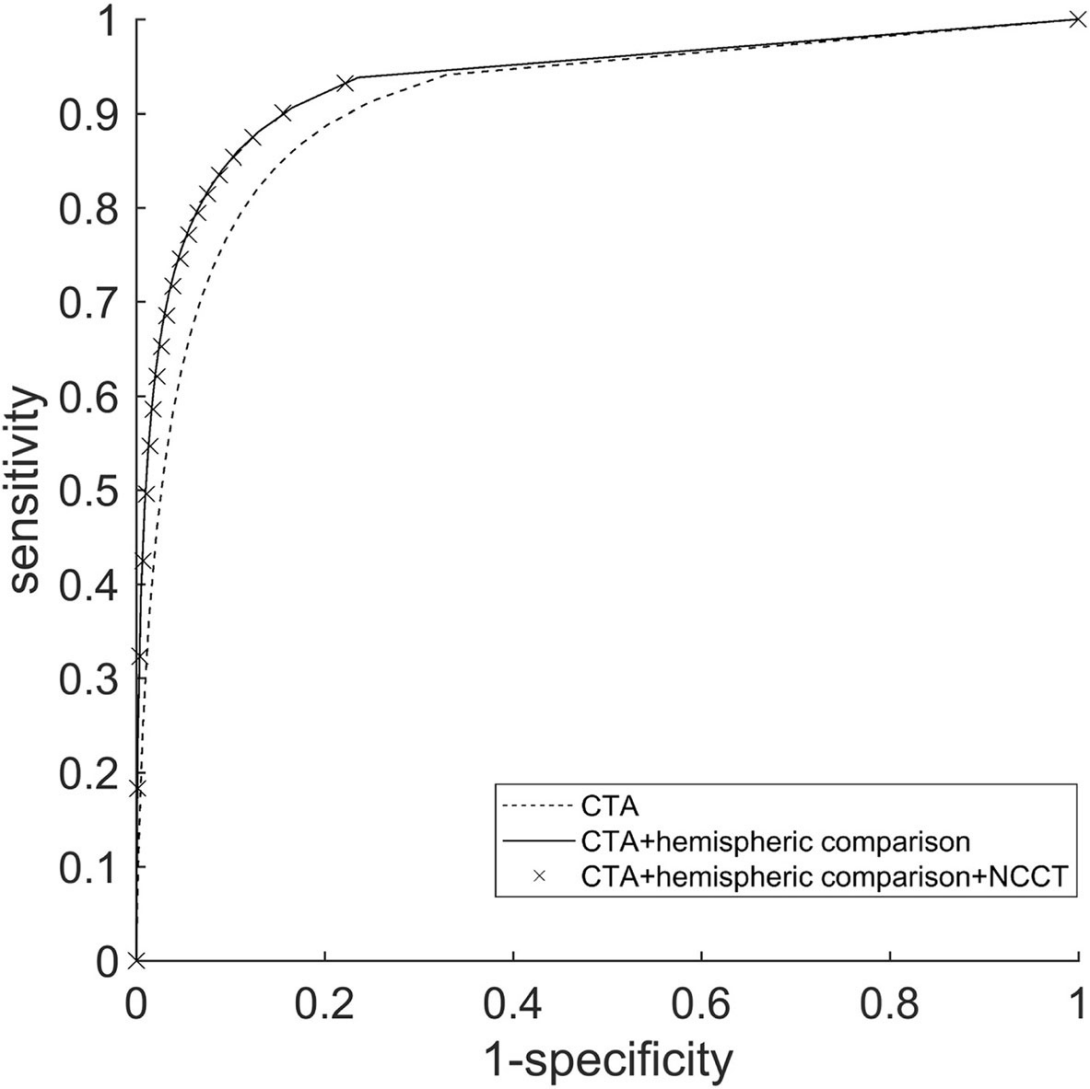
Voxel-wise receiver operating characteristic curves over the whole testing set were calculated for the three trained CNNs by varying the threshold value of the produced probability maps. An increase in performance can be seen when cerebral hemispheric comparison CTA was included in the CNN analysis

As explained in the “Methods” section, three separate CNNs were trained: (1) with CTA-only; (2) with CTA + cerebral hemispheric comparison; and (3) with CTA + cerebral hemispheric comparison + NCCT as inputs. Using a 0.5 threshold on probability maps voxel-wise, sensitivity was 0.67, 0.74, and 0.71; specificity 0.93, 0.96, and 0.96; and DSC 0.40, 0.55, and 0.55 for the three CNNs, respectively. The threshold was varied, showing the resulting ROC curves in Fig. 4. The ROC-AUC was 0.91, 0.93, and 0.93, respectively. These included all the voxels from the group A and B testing data. Variability between stroke patients was estimated from the group A testing data. The respective means ± standard deviation of the patient-wise ROC AUC was 0.89 ± 0.04, 0.92 ± 0.06, and 0.91 ± 0.05. A small improvement (*e.g.,* combined voxel-wise sensitivity increased by 0.07 and DSC by 0.15) was seen when the cerebral hemispheric comparison CTA was included, but there was practically no effect when the NCCT was added as a third input. The combination of CTA and NCCT was not investigated. The largest voxel-wise DSC value (0.61) was obtained with CTA + cerebral hemispheric comparison CTA inputs at 0.7 threshold.

During the retrospective visual investigation of the false positives, the appearance of unilateral and bilateral false positives in the CNN outputs (Table 2, Figs. 2 and 3) was linked with the age-related periventricular white matter hypoattenuation (see Fig. 3b, c). Cortical false positives were related to the widening of the cortical sulci due to cortical atrophy (see Fig. 3d). As a consequence, most of the false positives were located in the periventricular white matter and near to cortical cerebrospinal fluid spaces, to be associated with the normal aging effects on the brain.

**Fig. 5:**
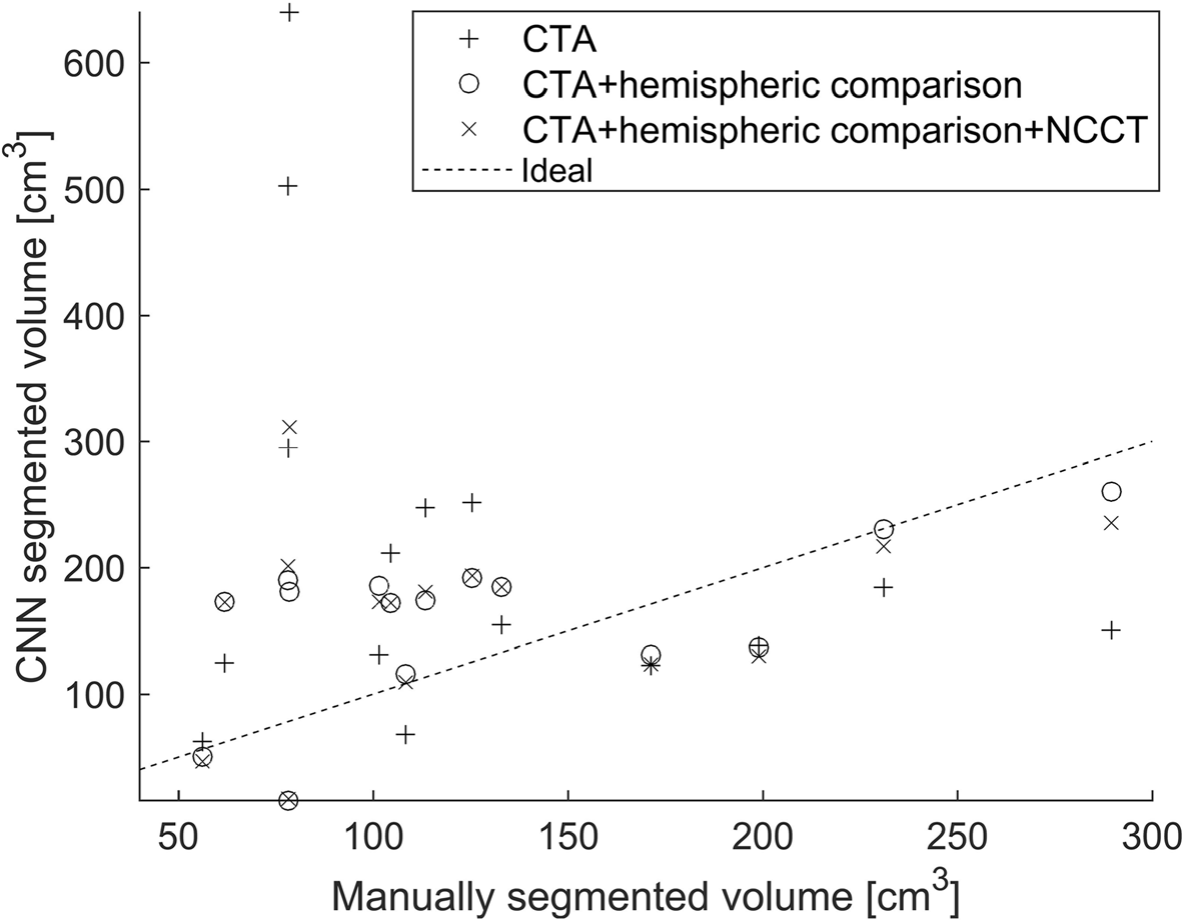
Stroke lesion volumes of the manual segmentation and three CNN outputs. The largest connected region is used in the volume calculation

Lesion volume comparisons between the manual delineations and CNN outputs (probability threshold 0.5) were calculated from the largest continuous lesion segments. The infarct lesion volumes in the manual segmentations varied from 11 to 293 cm^3^. The comparisons are shown in Fig. 5.

Neither manual interventions nor modifications had to be made for any of the volumes when visually verifying the image preprocessing. Training the CNNs using the 30 subjects took the total of 77 h for CTA-only, 122 h for CTA + cerebral hemispheric comparison-CTA, and 152 h for CTA + cerebral hemispheric comparison-CTA + NCCT input features.

The time for processing and CNN execution steps in an individual subject was as follows: 23 min for manual segmentation including data selection and saving; 2 min and 1 s for the file compression to save disk space; 3 min and 26 s for executing the CNN for CTA only; 3 min and 36 s for executing the CNN for CTA + cerebral hemispheric comparison; 3 min and 40 s executing the CNN for CTA + cerebral hemispheric comparison + NCCT features. This time included initialization and reading of the network weights, calculating outputs, and saving the results. Transfer from the picture archive, anonymization, and file type conversions was not included.

## Discussion

**Fig. 6:**
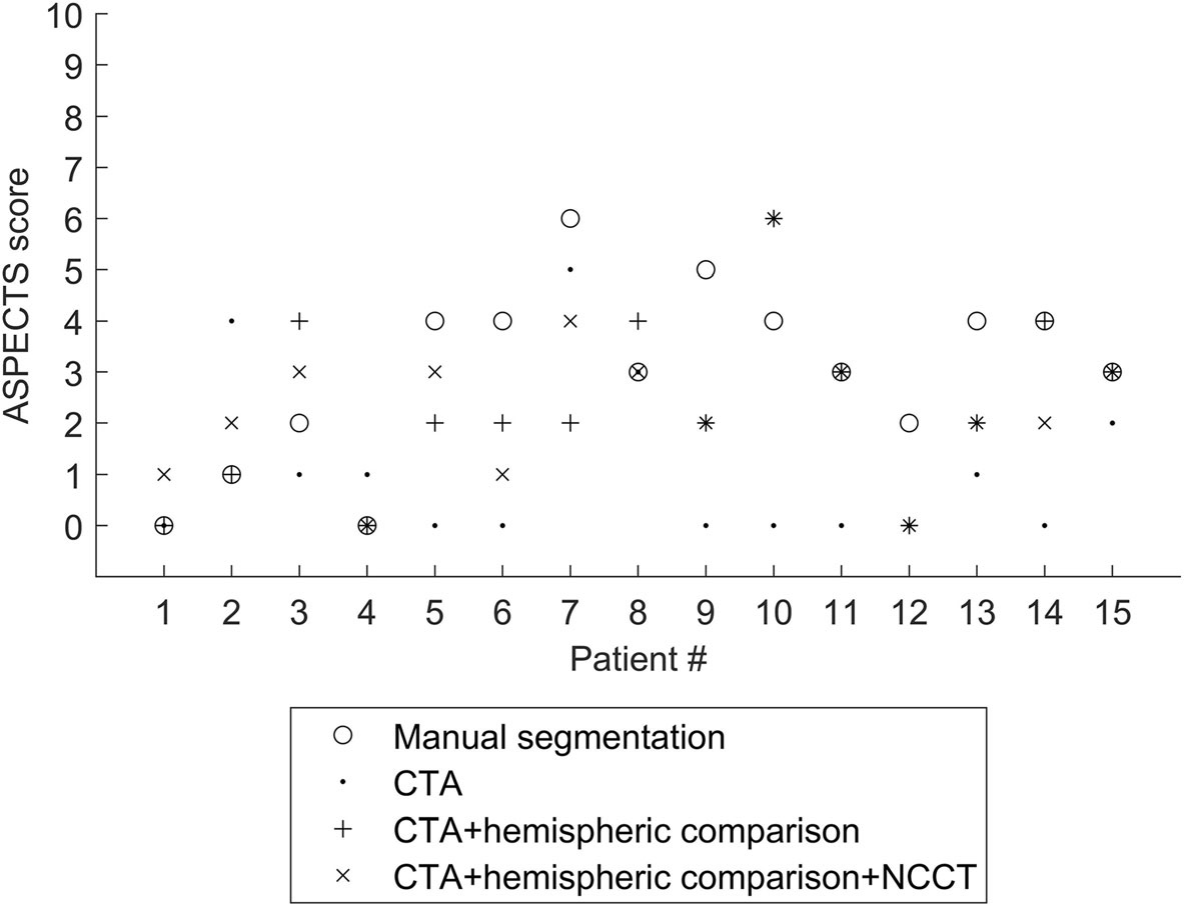
ASPECTS points of the manual segmentation and three CNN outputs for the group A patients (test data). All the scores are calculated from the CTA-SI; if a single positive voxel belongs to the specified region, it is marked as stroke positive. The number of positive regions is subtracted from 10 to get the ASPECTS points and the minimum (worst case) between the hemispheres is the reported value

The aim of this study was to investigate the feasibility of ischemic stroke detection from CTA-SI using 3D CNNs. All the lesions in the testing data were correctly lateralized, and a high correspondence to manual segmentations was achieved. Although only a minor effect was seen on the ROC-AUC values, cerebral hemispheric comparison CTA as an additional input was beneficial in improving the ischemic lesion detection specificity. A substantial increase in specificity can be seen when cerebral hemispheric comparison CTA was included as an input feature. A slight decrease in the number of false positives was seen when NCCT was included. No notable changes can be seen in sensitivity between the three outputs. A notable change was seen when the cerebral hemispheric comparison CTA was included and a very slight change for the worse with the addition of the NCCT input. A notable improvement to the average performance was seen when the cerebral hemispheric comparison CTA was included as an input feature. However, a general impression of overestimating small lesion volumes and underestimating large volumes compared to the manual segmentation was seen for all trained networks (Fig. 5). This can also be seen in the ASPECTS scores defined from the CTA-SI using manual segmentation and the three CNNs (Fig. 6): at low (manual) APSPECT scores (*i.e.,* large lesions), the CNN scores were overestimated and vice versa for higher manual ASPECTS scores (smaller more localized lesions).

CNNs have previously been applied in the ischemic stroke detection based on diffusion-weighted imaging (DWI) [24] and multi-spectral MRI [25]. Lisowska et al. [16] proposed using a CNN in dense vessel and ischemia detection from NCCT images. The ischemia detection ROC-AUC values were in line with the present study (0.92 and 0.93, respectively). Also in line was the usefulness of comparing hemispheres in the detection of ischemia. However, Lisowska et al. demonstrated a more substantial absolute increase in ROC-AUC (+ 0.18) than the present study (+ 0.02). Additionally, they included an anatomical context by atlas registration but for ischemia detection found it less important than the direct bilateral comparison. In order to include contralateral features and anatomical context, they designed a custom network architecture where left and right hemisphere features propagated in parallel layers. The current study demonstrated that hemisphere information can be successfully included by using a co-registered left-right flipped image as an additional data channel. This enabled the use of the 3D CNN framework originally designed for lesion detection from multi-channel MRI [17].

There are other architectural differences to the DeepMedic used in the present study. Primarily the CNN by Lisowska et al. [16] used a composition of 1D convolutions, whereas the DeepMedic is based on fully 3D convolutions. Maldjian et al. [26] and Nowinski et al. [27] have also utilized a cerebral hemispheric difference in their analysis workflows. However, they did not use CNNs. Sensitivity calculated from Maldjian et al. reported values for lentiform nucleus and insula (0.85) lower than the sensitivity calculated over the ASPECTS regions in the present study (0.93). Gillebert et al. [28] as well as Peter et al. [29] applied a texture and atlas-based approach to lesion segmentation. Gillebert et al. [28] found a slightly higher DSC (0.67) than the present study (0.61 at optimal threshold) for simulated data but reported high variance for performance using actual patient images. Peter et al. [29] reported a ROC AUC of 0.82 which was less than that of the present study. At least one infarct detection software has been made commercially available [30].

The above mentioned studies had NCCT images as inputs whereas the current study is the first one to apply a 3D CNN to CTA-SI. In the previous studies, difficulties in distinguishing between old and new stroke lesions, and age-related changes have been reported. Likewise, in this study, the areas with periventricular white matter hypoattenuation and cortical atrophy were mislabeled as stroke lesions. The severity of this problem was mitigated by the introduction of cerebral hemispheric comparison CTA, and to a lesser degree, NCCT in addition to hemispheric comparison CTA, as input features. However, bilateral ischemic strokes were not investigated. Detection of bilateral stroke might not benefit from the proposed cerebral hemispheric comparison method.

Volumetric diagnosis of the ischemic lesion on a CTA-SI *versus* DWI and CTP lesion volumes is a controversial topic. The studies using single- and 4-slice CT scanners showed a high correlation between CTA-SI and DWI lesion volume in acute stroke [31, 32]. CTA-SI acquired with a fast-acquisition protocol overestimates the volume of the infarct core compared to DWI. According to Yoo et al. [7], the lesion overestimation can be significant (> 25 cm3). Major disadvantage of the DWI is its limited availability in the emergency setting. On the other hand, the inclusion of CTP increases substantially the radiation exposure and the contrast agent doses. Our stroke protocol consisted of NCCT and CTP imaged before CTA test-bolus and CTA-SI. According to a recent review by Sharma et al. [8], despite the theoretic modeling suggesting CTA-SI to be predominantly blood-volume weighted, the more rapid modern CTA protocols may be too quick to achieve the arterial and tissue contrast steady-state. In addition, these protocols are more likely blood-flow-weighted.

The hypothesis of this study was also that CTA imaged precisely after the test-bolus the extent of ischemic core on CTA-SI is proportional to the cerebral blood flow (penumbra and core) deficit justifying the proposed deep learning approach. However, as can be seen in Fig. 5, there was a relatively high variance in the measured volumes between the manual and CNN segmentations, which makes the use of any definite volume thresholds problematic. One explaining factor for the discrepancy may be the objective difficulty of delineating the infarct region accurately. It may be partially responsible for the mismatch between the volumes for small lesions (whose volume was overestimated by CNN) and large lesions (whose volume was underestimated by CNN). On the other hand, the varying radiological manifestation of different infarct types may contribute to the neural network performance. This underlines the importance of using large training datasets.

The implementation of this study was primarily based on freely available open-source software and the steps could be combined into an automated stroke analysis pipeline. Low latency processing in a time critical situation where stroke imaging is usually applied is vital. The total analysis taking currently less than 6 min for an individual patient is approaching an acceptable level of delay. No optimization of network model or training parameters, *e.g.,* adjusting the learning rate, was performed for these results. It should also be noted that the time required for network transfers was not included in the estimates. Transfer delays could affect the total time significantly. Including the cerebral hemispheric comparison CTA as an input had only a miniscule effect on the processing time. DeepMedic implementation of a well-established Theano framework was chosen due to its previous success in multi-contrast MRI brain lesion segmentation challenge [25]. The present study showed a satisfactory performance for a different task and for different modality demonstrating the power of the network design in this “off-the-self” implementation.

The typical problem of model overfitting can be mitigated with a sufficiently representative selection of training data. These data should incorporate the variability in target manifestations as extensively as possible. The dataset comprised of different stroke severities, lesion volumes, symptom starting times, and various occluded arteries. This resulted in a small sampling of individual types. Stroke can manifest in multiple ways and therefore the small training set was a study limitation. Future improvements of the analysis workflow and larger data set are warranted for including the variety of the disease manifestations.

In addition, image resolution varied between data sets, which introduced additional variance. The effect was mitigated by using a unified resampling resolution. In a case of wide clinical application, the tool should remain robust against varying imaging parameters, including resolution. A similar limitation was the lack of thin slice NCCT. Although including the NCCT (along with CTA and hemispheric comparison CTA) had only a minor noticeable effect (small decrease in the false positive rate of non-infarcted patients), the authors believe that including both investigated additional feature inputs is a worthwhile pursuit. Moreover, NCCT alone or CTA + NCCT without hemispheric comparison were not investigated. Data processing could be further improved, by including atlas-based anatomical regions as described by Peter et al. [29] or by introducing additional features such as the requirement of hypoattenuation to extend onto the cortical surface. In addition, finding an optimum in the CNN parameter space, exploring alternative algorithms and implementations aimed at improving specificity and reducing processing time is essential when targeting the clinical deployment. Finally, a fully independent validation data from another imaging site and from a different vendor CT equipment should be used to assess the transferability and robustness of the trained network.

In conclusion, the detection of acute ischemic brain lesions by deep learning analysis of CTA-SI was studied and high sensitivity was achieved. Improvement in specificity was seen when contralateral hemisphere was included in the analysis. These encouraging findings demonstrate a potential of CNNs for a rapid, precise, and fully automated method to assist radiologist and clinicians in the detection of acute stroke lesions.

## Abbreviations

3D: Three-dimensional
ASPECTS: Alberta stroke program early CT scoring system
CNN: Convolutional neural network
CTA: Computed tomography angiography
CTA-SI: CTA source images
CTP: Computed tomography perfusion
DSC: Dice similarity coefficient
DWI: Diffusion-weighted imaging
NCCT: Non-contrast computed tomography
ROC AUC: Area under receiver operating characteristic curve
